# Examining Facets of Body Image Disturbance Across Anorexia Nervosa Illness and Recovery Stages

**DOI:** 10.1002/erv.70103

**Published:** 2026-04-01

**Authors:** Scarlett R. Croce, Amy C. Malcolm, Andrea Phillipou, Christina Ralph‐Nearman

**Affiliations:** ^1^ Centre for Mental Health Swinburne University of Technology Melbourne Victoria Australia; ^2^ Orygen Melbourne Victoria Australia; ^3^ Centre for Youth Mental Health The University of Melbourne Parkville Victoria Australia; ^4^ Orygen Specialist Program Parkville Youth Mental Health & Wellbeing Service Melbourne Victoria Australia; ^5^ Department of Psychological Sciences Swinburne University of Technology Melbourne Victoria Australia; ^6^ Department of Mental Health St Vincent’s Hospital Melbourne Victoria Australia; ^7^ Department of Mental Health Austin Hospital Melbourne Victoria Australia; ^8^ Palm Beach Atlantic University Health & Human Performance Palm Brach Florida USA

**Keywords:** anorexia nervosa, body dissatisfaction, body image disturbance, body perception, recovery stages

## Abstract

**Objective:**

Research suggests body image disturbance (BID) is a core feature of anorexia nervosa (AN), however, more research is required on how BID may differ across illness and recovery stages. This study aimed to investigate differences in BID facets (body image dissatisfaction and body image perception) among women with AN who were currently ill and at varying recovery stages, and women with no history of eating disorders (non‐ED controls).

**Method:**

One hundred ninety‐nine women (18–65 years old) meeting criteria for either current AN (AN‐C; *n* = 73), weight‐restoration (AN‐WR; *n* = 86), partial recovery (AN‐PR; *n* = 19), or full recovery (AN‐FR; *n* = 21), and a group of non‐ED controls (*n* = 128), completed online self‐report measures of BID and AN symptom severity.

**Results:**

The AN‐C and AN‐WR showed significantly worse body image dissatisfaction and body image perception than the AN‐FR and non‐ED control groups.

**Discussion:**

These findings suggest that BID differs across AN recovery stages and suggests that holistic recovery from AN includes the remediation of BID. The findings highlight the need for treatment to provide continuous support especially after the weight‐restoration period when BID may be at its peak.

## Highlights

- The findings of this study suggest that body image disturbance in AN differs across AN illness and recovery stages.
- The findings indicate that holistic recovery from AN may include the remediation of body image disturbance.
- The results suggest that support throughout the entire recovery process, especially during stages of increased body distress such after weight‐restoration, may be beneficial.

## 1 Introduction

Anorexia nervosa (AN) is a serious psychiatric illness which has among the highest risks of premature mortality of all mental health conditions (Birmingham et al. 2005; Mehler et al. 2022). Treatment outcomes for the disorder are poor with recovery rates of approximately 50%–60%, and relapse rates of up to 40% in the 1st year after treatment (Carter et al. 2012; Eddy et al. 2017; Keel et al. 2005; Murray et al. 2019; Steinhausen 2002; Walsh et al. 2021). These treatment outcomes highlight the need for further research into the driving factors of AN.

One potential driving factor in AN is body image disturbance (BID), defined as a disturbance in the way one’s body weight or shape is experienced (American Psychiatric Association 2013). BID is recognised as one of the main diagnostic criteria for AN, reflecting the high prevalence of BID in those with the disorder (Cash and Deagle 1997; Hagman et al. 2015), with researchers arguing that AN should be considered a body image disorder (Phillipou et al. 2018). Theoretical frameworks of AN suggest BID is a key causal factor of the disorder, involved in the onset and the maintenance of AN behaviours (e.g., Bruch 1981, 1982; Casper 1983; Garfinkel and Garner 1983; Goodsitt 1997; Oldershaw et al. 2019). Empirical research has established a strong connection between BID and AN, indicating BID is linked to the onset, maintenance and recovery from AN (e.g., Calugi and Dalle Grave 2019; Glashouwer et al. 2019; Junne et al. 2019; Ralph‐Nearman et al. 2021). For instance, research has indicated BID as a factor in AN relapse as it can lead to behavioural AN symptoms reemerging if not adequately addressed (Carter et al. 2004; Keel et al. 2005), and may also be an objective marker for illness severity (Ralph‐Nearman et al. 2021).

However, despite the centrality of BID in the presentation of AN and these research findings, there is a lack of consensus on the specific relationship between BID within AN. Some researchers have argued there is not enough current empirical evidence to suggest BID holds a causal role in AN maintenance or recovery, and instead they propose that BID should be considered a symptom of AN rather than a driving factor (e.g., Glashouwer et al. 2019; Munro et al. 2017; Schmidt and Treasure 2006; Steinglass and Walsh 2016). Further, the relationship between BID and AN recovery is currently unclear. Specifically, while treatments often focus on improving food intake and reducing other compensatory AN behaviours (such as overexercising or purging), research suggests BID remains persistent throughout treatment and post‐treatment (Benninghoven et al. 2006). More holistic definitions propose that recovery from AN goes beyond resolution of behaviours and weight restoration, and also includes psychological changes, including changes in how body image is experienced (i.e., a reduction in BID; e.g., Bardone‐Cone et al. 2018; Phillipou et al. 2025).

BID is a complex construct which is commonly thought to be comprised of several components, including cognitive/affective and perceptual components. Within the cognitive/affective component of BID, defined as attitudes and feelings held towards one's body (Glashouwer et al. 2019), multiple facets exist including body image dissatisfaction. Body image dissatisfaction refers to negative body beliefs and emotions (Cash and Smolak 2011; C. G. Fairburn et al. 2003), and has been strongly linked to AN (e.g., Cash and Deagle 1997; Hagman et al. 2015; Shagar et al. 2017; Stice et al. 2011). Meanwhile, the perpetual component of BID (which will be referred to as body image perception in this study) is defined as difficulties in correctly perceiving and judging body dimensions (Cash and Deagle 1997; Irvine et al. 2019). Examples of body image perception include bodily distortions such as objectively knowing one is thin but still feeling overweight and overestimating body size. Body image perception has received less research attention than body dissatisfaction, but existing studies have found a significant connection between this component of BID and AN (e.g., Cash and Deagle 1997; Farrell et al. 2005; Gardner and Brown 2014; Guardia et al. 2010; Hagman et al. 2015; Phillipou et al. 2016; Ralph‐Nearman et al. 2021; Smeets et al. 1997; Touyz et al. 1984).

Several gaps exist within the current BID and AN literature. First, research into BID and AN has primarily focused on body dissatisfaction. Specifically, researchers have argued that body dissatisfaction may not be holistically representative of BID due to being less severe in its impact on aspects of quality of life (e.g., daily functioning) compared to other BID facets (Cash et al. 2004). As such, it is critical that research investigate body image dissatisfaction along with other components of BID, such as body image perception, to gain a deeper understanding of the relationship between BID and AN recovery. Second, limited research within the AN literature has explored BID across different illness and recovery stages in clinical AN samples. In addition, existing research in BID and AN recovery has reported mixed results with some studies finding improvements in BID post‐treatment alongside other recovery markers such as weight gain (Calugi and Dalle Grave 2019; Dalhoff et al. 2019), while others did not (Junne et al. 2016). To our knowledge, no research to date has examined BID across various AN illness and recovery groups and a non‐ED control group.

As such, the aim of the current study was to assess differences in the BID facets of body dissatisfaction and body perception among five groups; individuals currently ill with AN (AN‐C), individuals who were weight restored but still experiencing behavioural and psychological symptoms (AN‐WR), individuals who were partially recovered from AN (AN‐PR), individuals who were fully recovered from AN (AN‐FR), and individuals with no history of any eating disorder (non‐ED controls). It was hypothesised that the AN‐C, AN‐WR, and AN‐PR groups would report significantly worse body dissatisfaction and body image perception than the AN‐FR group and non‐ED controls.

## 2 Methods

### 2.1 Participants

Participants were 327 women, aged between 18–65 years old (*M* = 29.49 and SD = 9.77) comprising five groups: AN‐C (*n* = 73), AN‐WR (*n* = 86), AN‐PR (*n* = 19), AN‐FR (*n* = 21 participants), and non‐ED Controls (*n* = 128). Table 1 displays demographic characteristics across recovery groups. A modified set of criteria from Bardone‐Cone et al. (2010) was used to allocate participants into one of these five groups (see Table 2); these modified criteria have been employed previously in other research (e.g., Croce et al. 2024). Specifically, four criteria were used to classify participants: (1) Reported diagnosis of AN, (2) presence of psychological ED symptoms, (3) frequency of behavioural ED symptoms, and (4) body mass index (BMI) score. Participants were asked if they had a current or past diagnosis of AN or any other eating disorder (ED). Presence of psychological ED symptoms was measured as a score greater than one standard deviation above community age‐matched norms on one or more subscale of the Eating Disorders Examination Questionnaire (EDE‐Q; please see Mond et al. 2004 for age‐specific norms for each EDE‐Q subscale). Frequency of behavioural symptoms were measured using the EDE‐Q questions on self‐induced vomiting, laxative use, fasting or binge eating, with behavioural symptoms counted as present if any of these behaviours were present over the last 28 days. Participant BMI was calculated based on self‐reported height and lowest adult weight over the past 3 months and compared to the 18.5 kg/m^2^ cut‐off indicative of clinically significant underweight.

**TABLE 1:** Comparison of demographic and clinical characteristics across groups.

	AN‐C	AN‐WR	AN‐PR	AN‐FR	Non‐ED controls	Whole sample	*F*	*p*	*η* ^2^
Age	27.68 (9.12)^a^	28.26 (8.92)^a^	29.68 (8.10)^a^	28.90 (8.81)^a^	31.41 (10.79)^a^	29.49 (9.77)	2.05	0.10	0.03
Ethnicity								0.09	
Aboriginal or Torres Strait Islander	4.1 (3)	3.5 (3)	0 (0)	0 (0)	0.8 (1)	2.1 (7)			
Caucasian	89.0 (65)	80.2 (69)	89.5 (17)	95.2 (20)	75.0 (96)	81.7 (267)			
Asian	4.1 (3)	4.7 (4)	5.3 (1)	0 (0)	13.3 (17)	7.6 (25)			
African	0 (0)	3.5 (3)	0 (0)	0 (0)	0.78 (1)	1.2 (4)			
Other	2.7 (2)	8.1 (7)	5.3 (1)	4.8 (1)	10.2 (13)	7.3 (24)			
BMI 3 months	16.00 (1.69)^a^	23.13 (4.62)^b^	22.08 (3.47)^b^	21.97 (3.22)^b^	23.35 (4.22)^b^			< 0.001	
EDE‐Q restraint	4.02 (1.59)^a^	3.73 (1.68)^a^	2.02 (1.09)^b^	0.52 (0.67)^c^	0.58 (0.71)^c^		130.95	< 0.001	0.62
EDE‐Q eating concern	3.58 (1.40)^a^	3.16 (1.31)^a^	1.84 (1.16)^b^	0.39 (0.44)^c^	0.29 (0.38)^c^		175.96	< 0.001	0.68
EDE‐Q shape concern	4.55 (1.48)^a^	4.74 (1.13)^a^	3.98 (1.11)^a^	1.36 (0.96)^b^	1.45 (1.00)^b^		150.60	< 0.001	0.65
EDE‐Q weight concern	4.33 (1.47)^a^	4.49 (1.20)^a^	3.87 (0.95)^a^	1.37 (0.90)^b^	1.23 (0.90)^b^		154.09	< 0.001	0.66
EDE‐Q global	4.12 (1.29)^a^	4.03 (1.16)^a^	2.93 (0.84)^b^	0.91 (0.63)^c^	0.89 (0.60)^c^		209.77	< 0.001	0.72

*Note:* Data reported as *M*(SD) or %(*n*) where relevant. Mean values not sharing the same superscript are significantly different at *p* < 0.05.

Abbreviations: AN = anorexia nervosa, AN‐C = currently ill AN, AN‐FR = fully recovered AN, AN‐PR = partially recovered AN, AN‐WR = weight‐restored AN, BMI 3 Months = lowest body mass index over the past 3 months, BMI = body mass index, ED = eating disorder, EDE‐Q = Eating disorder examination questionnaire, *F* = ANOVA *F* test reported, Non‐ED Controls = non‐eating disorder controls, *η* ^2^ = eta squared.

**TABLE 2:** Anorexia nervosa group criteria.

	AN‐C	AN‐WR^a^	AN‐PR	AN‐FR	Non‐ED controls
Self‐reported diagnosis of AN	Yes (current)	Yes (current)	Yes (current)	Yes (past)	No (incl. any other EDs)
Presence of psychological ED symptoms^b^	Yes	Yes	Yes	No	No
Presence of behavioural ED symptoms^b^	Yes	Yes	No	No	No
BMI below 18.5 kg/m^2^ over past 3 months	Yes	No	No	No	No

Abbreviations: AN = anorexia nervosa, AN‐C = currently ill AN, AN‐FR = fully recovered AN, AN‐PR = partially recovered AN, AN‐WR = weight‐restored AN, BMI = body mass index, ED = eating disorder, Non‐ED Controls = non‐eating disorder controls.

^a^ AN‐WR classification criteria for was created for this study. All other group classifications based on modified criteria from Bardone‐Cone et al. 2010.

^b^ As reported for the past 28 days.

Regarding the AN groups, Bardone‐Cone et al. (2010) initially outlined criteria for classifying AN illness and recovery groups into either active illness, partial recovery, and fully recovered. These criteria were used to guide allocation of participants in the current study. Additionally, we created a fourth AN classification group of *weight‐restored*. This group was used to categorise individuals who had a BMI above 18.5 kg/m^2^ but who had continuing behavioural ED symptoms, as these participants did not fit within the Bardone‐Cone et al. (2010) criteria for either the active illness or partial recovery groups. An additional modification was the use of the self‐report EDE‐Q instead of the clinician‐administered Eating Disorder Examination (EDE; as was used by Bardone‐Cone et al. 2010), due to the online nature of this study's data collection. The EDE‐Q has good concurrent validity and acceptable criterion validity with the EDE (Mond et al. 2004). As a result of this change, the timeframe of the behavioural ED symptoms criteria was also modified. Bardone‐Cone et al. (2010) outlined a 3 month timeframe for behavioural symptoms using the EDE, but we were only able to determine if behavioural symptoms had occurred in the past 28 days due to the timeframe EDE‐Q. Lastly, the AN‐PR group in the current study had a self‐reported diagnosis of current AN instead of no current diagnosis as in Bardone‐Cone et al. ’s (2010) recovery definition.

### 2.2 Procedure

The anonymous online survey was approved by the Swinburne University Human Research Ethics committee (SHR Project 2021/6021) and accessed via Qualtrics. Participants were recruited through Swinburne University of Technology's Research Experience Programme, participant registries held at Swinburne University, the University of Melbourne's student portal, advertisements posted through ED‐related organisations and websites, and public advertisements. After providing informed consent, participants completed demographic questions, reported psychiatric diagnoses (past and current), reported their height and weight over the past 3 months, and completed measures assessing ED symptoms and BID. As the survey was part of a larger study, additional measures were included and only measures relevant to the current research are reported here. A total of 1606 participants expressed interest and began the survey, though only 327 women were included from the larger sample (i.e., only women who completed the relevant measures, met inclusion criteria and were retained following data cleaning; please see Figure S1 for CONSORT diagram). The entire survey took approximately 30 min to complete and included a check for bot responses using reCAPTCHA V3. Data screening also involved reviewing qualitative responses in the dataset to check for repetitive or nonsensical answers. Participants were offered to enter a reimbursement lottery at the completion of the survey to receive one of five $100 AUD gift vouchers. The study was not pre‐registered.

### 2.3 Measures

#### 2.3.1 Female Body Scale (FBS)

The FBS is a self‐report measure of BID including body dissatisfaction and body perception. The FBS is a set of nine female figures ranging from emaciated to high adiposity presented according to a numerical scale ranging from a score of 1 (most emaciated) to 9 (most adipose). Participants were asked to select figures from the scale according to (1) Body Looks, that is, the way their current body *looks* to them, (2) Body Feel, that is, the way they *feel* in their current body, and (3) Body Ideal, that is, what their *ideal* body figure would be. Body dissatisfaction and body perception ratings were generated using the numerical values (‘scores’) of participants' chosen figures. Body dissatisfaction scores were calculated using the following formula: ideal body score *minus* body look score. Body perception ratings were generated using the following formula: body look score *minus* body feel score. Body dissatisfaction and perception scores range from −8 to +8, with scores closer to zero indicating less body image dissatisfaction and better body perception, respectively. Greater negative scores indicate greater body image dissatisfaction towards an idealised thin body, and poorer body image perception whereby individuals feel larger than they think they look. Construct validity for the scales are good, with strong associations to actual body measurements and the EDE‐Q (Ralph‐Nearman and Filik 2020). Test‐retest reliability is also good (*p <* 0.001 over a one to 3 week period for the FBS; Ralph‐Nearman and Filik 2020).

#### 2.3.2 Eating Disorders Examination Questionnaire (EDE‐Q)

The EDE‐Q 6.0 is a self‐report measure comprising 28 items, designed to assess the frequency and severity of ED behaviours and psychopathology over the last 28 days. The EDE‐Q uses a 7‐point Likert scale and is divided into four subscales: restraint, eating concern, shape concern, and weight concern, in addition to a global score that is derived by averaging the four subscales (C. Fairburn and Beglin 2008). Both the global score and the subscale scores range from zero to six, with elevated scores signifying worse ED symptomatology. Moreover, six items are dedicated to assessing the frequency of ED behaviours over the past 28 days, which are not included in the subscale scoring. For the present study, items that measure the frequency of behaviours such as self‐induced vomiting, laxative use, fasting, and binge‐eating were employed to classify AN groups, as outlined in the participant section. The EDE‐Q has demonstrated high internal consistency across each subscale, with alpha coefficients of 0.84, 0.93, 0.89, and 0.78 for restraint, shape concern, weight concern, and eating concern, respectively. In the current study, internal consistency for the global score was excellent (*α* = 0.97), while the subscales exhibited good to excellent consistency (*α* = 0.91, 0.90, 0.94, and 0.89, respectively as above). Test‐retest reliability is also high for all subscales (*p <* 0.001 over a 2 week period; Luce and Crowther 1999).

### 2.4 Statistical Analysis

Statistical analyses were conducted using SPSS (Version 30; IBM Corp 2024). To address the hypotheses predicting differences in body dissatisfaction and body perception between groups (AN‐C, AN‐WR, AN‐PR, AN‐FR, and non‐ED controls), FBS body dissatisfaction and perception scores were compared between groups using 10 independent sample *t*‐tests for each variable as due to significant assumption violations including lack of homogeneity, ANOVAs were deemed inappropriate for the analyses. Bonferroni corrections were utilised for both sets of 10 independent sample *t*‐tests, with an adjusted α‐level of (0.05/20) = 0.003. Effect sizes were measured using Hedge's *g* due violations to Levene’s test for equality of variance and small group sizes (*g* = 0.02 indicates a small effect, *g* = 0.05 indicates a medium effect, and *g* ≥ 0.08 indicates a large effect; Cohen 2013). Group comparisons for participant demographics were conducting using a one‐way analysis of variance (ANOVA) to analyse age and chi‐squared tests for ethnicity. Clinical characteristics (BMI and EDE‐Q) were compared between groups using one‐way ANOVAs. An a priori power analysis indicated that 64 participants per group was required to achieve adequate power for our primary analyses. This sample size was not achieved, so Bonferroni corrections and Hedge's *g* were employed in an attempt to address this issue.

## 3 Results

Groups did not significantly differ on age or ethnicity. As expected, groups significantly differed on both BMI and EDE‐Q scores, with the lowest BMI and greatest EDE‐Q scores in AN‐C (see Table 1).

**FIGURE 1:**
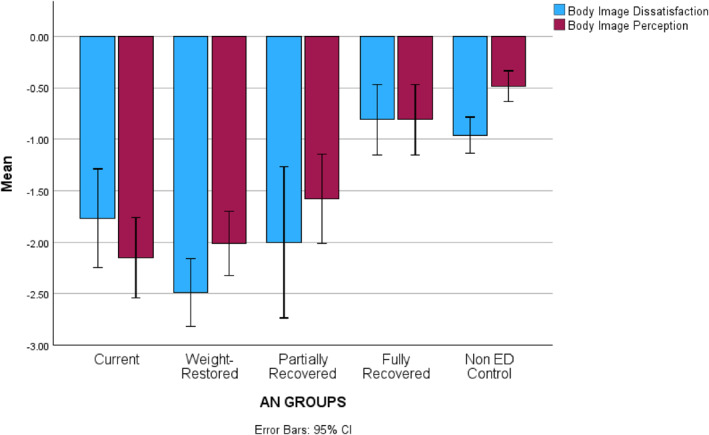
Mean scores for body image dissatisfaction and body image perception. Scores closer to 0 indicate lower levels of body image dissatisfaction and body image perception.

Results for the group comparisons are presented in Table 3 and Figure 1. Independent *t*‐tests showed that both body dissatisfaction and body perception were significantly poorer in the AN‐C group as compared to AN‐FR and non‐ED controls; in AN‐WR as compared to AN‐FR and non‐ED controls; and in the AN‐PR group as compared to non‐ED controls. The AN‐PR group did not significantly differ to AN‐FR on body perception or body dissatisfaction. No significant differences were found on body dissatisfaction or body perception between the AN‐C, AN‐WR, and AN‐PR groups, or between AN‐FR and non‐ED controls.

**TABLE 3:** Comparisons of body image dissatisfaction and body image perception across groups.

		AN‐C	AN‐WR	AN‐PR	AN‐FR	Non‐ED controls
		*t* (*g*)	*t* (*g*)	*t* (*g*)	*t* (*g*)	*t* (*g*)
Body dissatisfaction	AN‐C	—				
	AN‐WR	2.54 (0.40)	—			
	AN‐PR	0.46 (0.12)	−1.26 (−0.32)	—		
	AN‐FR	−3.30^a^ (−0.51)	−7.24^a^ (−1.18)	−3.18 (−0.99)	—	
	Non‐ED controls	−3.15^a^ (−0.55)	−8.19^a^ (−1.23)	−3.92^a^ (−0.96)	0.66 (0.16)	—
Body perception	AN‐C	—				
	AN‐WR	−0.56 (−0.9)	—			
	AN‐PR	−1.44 (−0.37)	−1.24 (−0.31)	—		
	AN‐FR	−5.27^a^ (−0.88)	−5.30^a^ (−0.88)	−2.95 (−0.93)	—	
	Non‐ED controls	−7.97^a^ (−1.37)	−8.74^a^ (−1.34)	−5.14^a^ (−1.26)	−1.63 (0.11)	—

Abbreviations: AN‐C = currently ill AN, AN‐FR = fully recovered AN, AN‐PR = partially recovered AN, AN‐WR = weight‐restored AN, *g* = Hedge's correction point estimate, Non‐ED Controls = non‐eating disorder controls, *t* = *t*‐score.

^a^ *p* values < 0.003 which are significant using the Bonferroni correction.

## 4 Discussion

The aim of this study was to examine differences in body dissatisfaction and body perception between AN groups during active illness and stages of recovery, as well as in non‐ED controls. In partial support of our hypothesis, body image dissatisfaction and body image perception were found to be worse in AN‐C and AN‐WR groups, compared to AN‐FR and non‐ED control groups. However, while the AN‐PR group showed worse body image dissatisfaction and body image perception compared to the non‐ED controls, they did not significantly differ from the AN‐FR group.

The findings of the current study indicate BID facets may differ across AN illness stages, suggesting that changes in both body dissatisfaction and body perception may potentially be important factors during AN recovery. The findings align with previous research which observed reductions in BID throughout AN recovery (e.g., Calugi and Dalle Grave 2019; Dalhoff et al. 2019). However, the literature on BID in AN recovery is mixed and as such, our results did not align with other studies which found no significant changes in BID over the course of recovery (e.g., Junne et al. 2016). The disparity between these findings may be due to the specific facet of BID that were examined. As BID is a broad multidimensional concept, different facets of BID may have varying relationships with AN recovery. While the current study found that body dissatisfaction and body perception had a similar pattern across recovery groups, it may be that other facets react differently over the course of recovery. As such, it is recommended that more large‐scale studies are conducted assessing multiple aspects of BID in order to examine the nuanced relationship between BID and AN to ensure a better understanding of this complex factor.

Results also showed that fully recovered individuals did not significantly differ to non‐ED controls on either body image dissatisfaction or body image perception. This finding suggests those who are fully recovered from AN may have non‐clinical levels of BID similar to those who have never experienced an ED. As such, this finding implies that individuals who are fully recovered from AN may potentially experience a significant shift in the ways they perceive and experience their bodies, a factor which may be central to achieving full recovery. This finding also provides evidence against the notion that people with a history of AN will always have persistent BID even when recovered, a common notion when using behavioural and weight‐based definitions of recovery. This implication may be significant as it could partially explain the high relapse rates seen in AN (Carter et al. 2012; Keel et al. 2005). By using the behavioural and weight‐based definitions, many individuals may only achieve a form of ‘pseudo recovery’ (e.g., physical recovery without psychological change), and thus be at high risk of relapsing (Sibeoni et al. 2017).

Additionally, our results indicated that individuals who were weight‐restored did not significantly differ from those currently ill with AN on either facet of BID (body dissatisfaction or body perception). This finding shows that weight‐restored individuals may experience clinically significant levels of BID similar to those of currently ill individuals, indicating that BID may not automatically reduce once weight restoration has occurred. This result further suggests weight‐restoration alone might not be a sufficient metric of recovery, an implication which will be expanded upon below.

Results of the current study also indicate that partially recovered individuals did not differ significantly from currently ill, weight‐restored or fully recovered individuals on either BID facet. This finding may be due to the nature of the partially recovered group sitting ‘in between’ recovery states, due to being weight‐restored and having ceased AN behaviours but still experiencing psychological symptoms. As such, it may that this group is less homogeneous and distinctive compared to the other recovery groups, which can be seen in the large variability of scores within this group.

Findings of the current study hold possible implications for how those currently ill with AN experience BID. Specifically, it was found that the currently ill group experienced less body dissatisfaction compared to the weight‐restored and partially recovered groups. Those currently ill with AN may experience less body dissatisfaction due to being in an ego‐syntonic state (i.e., a state which aligns with their personal goals) as they are at a low weight and are actively engaging in AN behaviours. Moreover, weight‐restored individuals may score the highest in body dissatisfaction due to experiencing weight gain and having lingering psychological and behavioural symptoms, resulting in substantial body image distress. Conversely, currently ill individuals had the poorest body perception; poorer than both weight‐restored and partially recovered individuals. As such, while currently ill individuals may have less body dissatisfaction they still may experience a stronger disconnect between their visually perceived body and their ‘felt’ perception of their body, which may be connected to poorer interoceptive processes in the ill state (e.g., Phillipou et al. 2022). Collectively, these findings suggest BID may be a key aspect of AN linked to both acute illness and recovery states.

### 4.1 Clinical Implications

The findings of this study hold several potential implications for AN treatment. First, findings emphasise that continuing treatment across different stages of recovery, especially after weight‐restoration when body dissatisfaction appears to be heightened, may be important. As mentioned, results found that weight‐restored individuals had the greatest degree of body dissatisfaction compared to all other groups. Recovery is reported by individuals with lived experience to be highly distressing (e.g., Broomfield et al. 2021; Lamoureux and Bottorff 2005; Williams et al. 2016). This distress may be in part due to increased weight gain while being unable to rely on ED behaviours to alleviate distress. In addition, as body dissatisfaction has been linked to increased risk of relapse in AN (Carter et al. 2004; Keel et al. 2005), it may be that weight‐restored individuals are at the highest risk of relapse. As such, treatment should ensure continuity of care throughout recovery with increased support provided for weight‐restored individuals during this critical period of recovery where relapse is potentially more likely due to higher levels of body dissatisfaction.

Second, the findings of the current study also suggest that treatment should endeavour to assess multiple facets of BID as different facets may be more relevant at different points of treatment. Results showed body image dissatisfaction was poorest for those who were weight‐restored, however, body image perception was poorest for currently ill individuals. Assessing these different facets of BID is important as these may influence recovery. For example, it may be beneficial for treatment to focus on perceptual BID at the beginning of treatment, whereas cognitive/affective BID may need to be prioritised during the weight‐restoration stage. In addition, these findings suggest that treatments which specifically address BID may be beneficial for individuals with AN. For instance, treatments such as enhanced cognitive behavioural therapy (CBT‐E) which address the behavioural components of BID such as body checking (Murphy et al. 2010), might be recommended along with new modalities such as the use of virtual reality (VR) which aims to specifically address the cognitive/affective and perceptual aspects of BID in EDs (Riva et al. 2021).

Finally, the findings provide support for moving away from purely behavioural and weight‐based definitions of recovery, and suggest that more holistic recovery that includes resolution of psychological symptoms is important (Phillipou et al. 2025). Currently, many of the common treatments for AN such as family‐based therapy (FBT) focus primarily on the physical symptoms of the illness such as low weight (Rienecke 2017) and do not specifically address BID. This mismatch in treatment priorities may be linked to the high relapse rates for those with AN (Carter et al. 2012; Keel et al. 2005). As such, focussing on achieving more holistic recovery, including the resolution of BID—perhaps through techniques such as embodiment (i.e., a sense of being in touch with one's body)—may help reduce the current high rates of relapse.

### 4.2 Strengths and Limitations

To our knowledge this is the first study to examine facets of BID across different AN illness and recovery states, allowing us to gain a deeper understanding of how BID may function across illness and recovery stages. In addition, the current study has the strength of large sample size ensuring adequate power to detect group differences and has expanded our understanding of AN recovery stages by building upon the comprehensive recovery criteria established by Bardone‐Cone et al. (2010). The current study also has some key limitations, such as the causal role of BID in AN recovery remaining unclear due to the cross‐sectional nature of the study. Future research would benefit from undertaking longitudinal research that examines multiple facets of BID. Of note, recovery is more dynamic than a linear trajectory from illness to full recovery, and future longitudinal research should aim to better understand BID across real‐world illness trajectories. Further, additional qualitative research on patients’ experiences should be undertaken to enrich our understanding of the relationship between BID and AN recovery.

Additionally, despite the total sample size for the present study being substantial at 327 participants, the sample size for some AN groups were smaller which may have influenced the statistical power for certain comparisons (e.g., between the AN‐PR and AN‐FR groups who had 19 and 21 participants, respectively). Furthermore, it is important to note that while this study aimed to build upon Bardone‐Cone et al. (2010) by adding a AN‐WR group, the focus on BMI alone as a metric of recovery is a limitation as it lacks corroboration with other indices of recovery. Finally, the self‐report nature of the study is an important limitation to note as the categorisation of AN groups relied on self‐reported information (including self‐reported AN diagnosis, height and weight) that could not be verified by the researchers.

## 5 Conclusion

The findings of the current study deepen our understanding of the role of BID in AN by demonstrating differences in BID facets across illness and recovery stages. The findings suggest that BID may be a core feature of the disorder, potentially linked to recovery and as such, should be assessed and targeted throughout treatment. The findings further highlight the need for increased support during critical stages of recovery such as weight‐restoration when BID may be at its highest.

## Author Contributions

**Scarlett R. Croce:** conceptualization, data curation, formal analysis, investigation, methodology, project administration, writing – original draft, writing – review and editing. **Amy C. Malcolm:** conceptualization, data curation, formal analysis, methodology, resources, supervision, validation, visualization, writing – review and editing. **Andrea Phillipou:** conceptualization, methodology, project administration, resources, supervision, validation, visualization, writing – review and editing. **Christina Ralph‐Nearman:** conceptualization, methodology, resources, supervision, validation, visualization, writing – review and editing.

## Funding

Scarlett R. Croce is supported by Swinburne University Postgraduate Research Award (SUPRA). A/Prof Andrea Phillipou and Dr Amy Malcolm were supported by a National Health and Medical Research Council (NHMRC) Project Grant (CIA‐GNT1159953).

## Conflicts of Interest

The authors declare no conflicts of interest.

## Supporting information


**Figure S1:** CONSORT 2025 Flow Diagram.

## Data Availability

The data can be requested from the corresponding author upon reasonable request.

## References

[erv70103-bib-0001] American Psychiatric Association . 2013. Diagnostic and Statistical Manual of Mental Disorders. 5th ed. American Psychiatric Association.

[erv70103-bib-0002] Bardone‐Cone, A. M. , M. B. Harney , C. R. Maldonado , et al. 2010. “Defining Recovery From an Eating Disorder: Conceptualization, Validation, and Examination of Psychosocial Functioning and Psychiatric Comorbidity.” Behaviour Research and Therapy 48, no. 3: 194–202. 10.1016/j.brat.2009.11.001. 19945094 PMC2829357

[erv70103-bib-0003] Bardone‐Cone, A. M. , R. A. Hunt , and H. J. Watson . 2018. “An Overview of Conceptualizations of Eating Disorder Recovery, Recent Findings, and Future Directions.” Current Psychiatry Reports 20, no. 9: 79. 10.1007/s11920-018-0932-9. 30094740

[erv70103-bib-0004] Benninghoven, D. , E. Jürgens , A. Mohr , I. Heberlein , S. Kunzendorf , and G. Jantschek . 2006. “Different Changes of Body‐Images in Patients With Anorexia or Bulimia Nervosa During Inpatient Psychosomatic Treatment.” European Eating Disorders Review 14, no. 2: 88–96. 10.1002/erv.670.

[erv70103-bib-0005] Birmingham, C. L. , J. Su , J. A. Hlynsky , E. M. Goldner , and M. Gao . 2005. “The Mortality Rate From Anorexia Nervosa.” International Journal of Eating Disorders 38, no. 2: 143–146. 10.1002/eat.20164. 16134111

[erv70103-bib-0006] Broomfield, C. , P. Rhodes , and S. Touyz . 2021. “How and Why Does the Disease Progress? A Qualitative Investigation of the Transition Into Long‐Standing Anorexia Nervosa.” Journal of Eating Disorders 9, no. 1: 103. 10.1186/s40337-021-00458-w. 34404490 PMC8371900

[erv70103-bib-0007] Bruch, H. 1981. “Developmental Considerations of Anorexia Nervosa and Obesity.” Canadian Journal of Psychiatry 26, no. 4: 212–217. 10.1177/070674378102600402. 7296431

[erv70103-bib-0008] Bruch, H. 1982. “Anorexia Nervosa: Therapy and Theory.” American Journal of Psychiatry 139, no. 12: 1531–1538. 10.1176/ajp.139.12.1531. 6816075

[erv70103-bib-0009] Calugi, S. , and R. Dalle Grave . 2019. “Body Image Concern and Treatment Outcomes in Adolescents With Anorexia Nervosa.” International Journal of Eating Disorders 52, no. 5: 582–585. 10.1002/eat.23031. 30702170

[erv70103-bib-0010] Carter, J. C. , E. Blackmore , K. Sutandar‐Pinnock , and D. B. Woodside . 2004. “Relapse in Anorexia Nervosa: A Survival Analysis.” Psychological Medicine 34, no. 4: 671–679. 10.1017/s0033291703001168. 15099421

[erv70103-bib-0011] Carter, J. C. , K. B. Mercer‐Lynn , S. J. Norwood , et al. 2012. “A Prospective Study of Predictors of Relapse in Anorexia Nervosa: Implications for Relapse Prevention.” Psychiatry Research 200, no. 2–3: 518–523. 10.1016/j.psychres.2012.04.037. 22657951

[erv70103-bib-0012] Cash, T. F. , and E. A. Deagle 3rd. 1997. “The Nature and Extent of Body‐Image Disturbances in Anorexia Nervosa and Bulimia Nervosa: A Meta‐Analysis.” International Journal of Eating Disorders 22, no. 2: 107–125. 10.1002/(sici)1098-108x(199709)22:2<107::aid-eat1>3.3.co;2-y. 9261648

[erv70103-bib-0013] Cash, T. F. , K. A. Phillips , M. T. Santos , and J. I. Hrabosky . 2004. “Measuring ‘Negative Body Image’: Validation of the Body Image Disturbance Questionnaire in a Nonclinical Population.” Body Image 1, no. 4: 363–372. 10.1016/j.bodyim.2004.10.001.

[erv70103-bib-0014] Cash, T. F. , and L. Smolak . 2011. Body Image: A Handbook of Science, Practice, and Prevention, 2nd ed. Guilford Press.

[erv70103-bib-0015] Casper, R. C. 1983. “Some Provisional Ideas Concerning the Psychologic Structure in Anorexia Nervosa and Bulimia.” In Anorexia Nervosa: Recent Developments in Research, Vol. xvii, edited by P. L. Darby , 387–392. A.R. Liss.

[erv70103-bib-0016] Cohen, J. 2013. Statistical Power Analysis for the Behavioral Sciences. Routledge.

[erv70103-bib-0017] Croce, S. , A. Malcolm , C. Ralph‐Nearman , and A. Phillipou . 2024. “Examining Identity Functioning in Anorexia Nervosa Across Illness and Recovery Stages.” International Journal of Eating Disorders 57, no. 9: 1959–1968. 10.1002/eat.24247. 38995092

[erv70103-bib-0018] Dalhoff, A. W. , H. Romero Frausto , G. Romer , and I. Wessing . 2019. “Perceptive Body Image Distortion in Adolescent Anorexia Nervosa: Changes After Treatment.” Frontiers in Psychiatry 10: 748. 10.3389/fpsyt.2019.00748. 31681048 PMC6803517

[erv70103-bib-0019] Eddy, K. T. , N. Tabri , J. J. Thomas , et al. 2017. “Recovery From Anorexia Nervosa and Bulimia Nervosa at 22‐Year Follow‐Up.” Journal of Clinical Psychiatry 78, no. 2: 184–189. 10.4088/jcp.15m10393. 28002660 PMC7883487

[erv70103-bib-0020] Fairburn, C. , and S. Beglin . 2008. “Eating Disorder Examination Questionnaire (EDE‐Q 6.0).” In Cognitive Behavior Therapy and Eating Disorders, 309–313. 10.1037/t03974-000.

[erv70103-bib-0021] Fairburn, C. G. , Z. Cooper , and R. Shafran . 2003. “Cognitive Behaviour Therapy for Eating Disorders: A ‘Transdiagnostic’ Theory and Treatment.” Behaviour Research and Therapy 41, no. 5: 509–528. 10.1016/s0005-7967(02)00088-8. 12711261

[erv70103-bib-0022] Farrell, C. , M. Lee , and R. Shafran . 2005. “Assessment of Body Size Estimation: A Review.” European Eating Disorders Review 13, no. 2: 75–88. 10.1002/erv.622.

[erv70103-bib-0024] Gardner, R. M. , and D. L. Brown . 2014. “Body Size Estimation in Anorexia Nervosa: A Brief Review of Findings From 2003 Through 2013.” Psychiatry Research 219, no. 3: 407–410. 10.1016/j.psychres.2014.06.029. 25023364

[erv70103-bib-0025] Garfinkel, P. , and D. Garner . 1983. “The Multidetermined Nature of Anorexia Nervosa.” In Anorexia Nervosa: Recent Developments in Research, edited by P. L. Darby , P. E. Garfinkel , D. M. Garner , and D. V. Coscina , 3–14. Alan R. Liss.

[erv70103-bib-0026] Glashouwer, K. A. , R. M. L. Van Der Veer , F. Adipatria , P. J. De Jong , and S. Vocks . 2019. “The Role of Body Image Disturbance in the Onset, Maintenance, and Relapse of Anorexia Nervosa: A Systematic Review.” Clinical Psychology Review 74: 101771. 10.1016/j.cpr.2019.101771. 31751876

[erv70103-bib-0027] Goodsitt, A. 1997. “Eating Disorders: A Self‐Psychological Perspective.” In Handbook of Treatment for Eating Disorders. 2nd ed., 205–228. Guilford Press.

[erv70103-bib-0028] Guardia, D. , G. Lafargue , P. Thomas , V. Dodin , O. Cottencin , and M. Luyat . 2010. “Anticipation of Body‐Scaled Action Is Modified in Anorexia Nervosa.” Neuropsychologia 48, no. 13: 3961–3966. 10.1016/j.neuropsychologia.2010.09.004. 20833193

[erv70103-bib-0029] Hagman, J. , R. M. Gardner , D. L. Brown , J. Gralla , J. M. Fier , and G. K. W. Frank . 2015. “Body Size Overestimation and Its Association With Body Mass Index, Body Dissatisfaction, and Drive for Thinness in Anorexia Nervosa.” Eating and Weight Disorders ‐ Studies on Anorexia, Bulimia and Obesity 20, no. 4: 449–455. 10.1007/s40519-015-0193-0. 25929983

[erv70103-bib-0030] IBM Corp . 2024. “IBM.” In IBM SPSS Statistics for Windows (Version 30.0).

[erv70103-bib-0031] Irvine, K. R. , K. McCarty , K. J. McKenzie , et al. 2019. “Distorted Body Image Influences Body Schema in Individuals With Negative Bodily Attitudes.” Neuropsychologia 122: 38–50. 10.1016/j.neuropsychologia.2018.11.015. 30500663

[erv70103-bib-0032] Junne, F. , B. Wild , G. Resmark , et al. 2019. “The Importance of Body Image Disturbances for the Outcome of Outpatient Psychotherapy in Patients With Anorexia Nervosa: Results of the ANTOP‐Study.” European Eating Disorders Review 27, no. 1: 49–58. 10.1002/erv.2623. 30009554

[erv70103-bib-0033] Junne, F. , S. Zipfel , B. Wild , et al. 2016. “The Relationship of Body Image With Symptoms of Depression and Anxiety in Patients With Anorexia Nervosa During Outpatient Psychotherapy: Results of the ANTOP Study.” Psychotherapy (Chic) 53, no. 2: 141–151. 10.1037/pst0000064. 27267500

[erv70103-bib-0034] Keel, P. K. , D. J. Dorer , D. L. Franko , S. C. Jackson , and D. B. Herzog . 2005. “Postremission Predictors of Relapse in Women With Eating Disorders.” American Journal of Psychiatry 162, no. 12: 2263–2268. 10.1176/appi.ajp.162.12.2263. 16330589

[erv70103-bib-0035] Lamoureux, M. M. H. , and J. L. Bottorff . 2005. “‘Becoming the Real Me’: Recovering From Anorexia Nervosa.” Health Care for Women International 26, no. 2: 170–188. 10.1080/07399330590905602. 15804915

[erv70103-bib-0036] Luce, K. H. , and J. H. Crowther . 1999. “The Reliability of the Eating Disorder Examination—Self‐Report Questionnaire Version (EDE‐Q).” International Journal of Eating Disorders 25, no. 3: 349–351. 10.1002/(sici)1098-108x(199904)25:3<349::aid-eat15>3.0.co;2-m. 10192002

[erv70103-bib-0037] Mehler, P. S. , A. Watters , T. Joiner , and M. J. Krantz . 2022. “What Accounts for the High Mortality of Anorexia Nervosa?” International Journal of Eating Disorders 55, no. 5: 633–636. 10.1002/eat.23664. 34997783

[erv70103-bib-0038] Mond, J. M. , P. J. Hay , B. Rodgers , C. Owen , and P. J. Beumont . 2004. “Validity of the Eating Disorder Examination Questionnaire (EDE‐Q) in Screening for Eating Disorders in Community Samples.” Behaviour Research and Therapy 42, no. 5: 551–567. 10.1016/s0005-7967(03)00161-x. 15033501

[erv70103-bib-0039] Munro, C. , L. Randell , and S. M. Lawrie . 2017. “An Integrative Bio‐Psycho‐Social Theory of Anorexia Nervosa.” Clinical Psychology & Psychotherapy 24, no. 1: 1–21. 10.1002/cpp.2047. 27739190

[erv70103-bib-0040] Murphy, R. , S. Straebler , Z. Cooper , and C. G. Fairburn . 2010. “Cognitive Behavioral Therapy for Eating Disorders.” Psychiatric Clinics of North America 33, no. 3: 611–627. 10.1016/j.psc.2010.04.004. 20599136 PMC2928448

[erv70103-bib-0041] Murray, S. B. , D. S. Quintana , K. L. Loeb , S. Griffiths , and D. Le Grange . 2019. “Treatment Outcomes for Anorexia Nervosa: A Systematic Review and Meta‐Analysis of Randomized Controlled Trials.” Psychological Medicine 49, no. 4: 535–544. 10.1017/S0033291718002088. 30101734

[erv70103-bib-0042] Oldershaw, A. , H. Startup , and T. Lavender . 2019. “Anorexia Nervosa and a Lost Emotional Self: A Psychological Formulation of the Development, Maintenance, and Treatment of Anorexia Nervosa.” Frontiers in Psychology 10: 219. 10.3389/fpsyg.2019.00219. 30886593 PMC6410927

[erv70103-bib-0043] Phillipou, A. , D. J. Castle , and S. L. Rossell . 2018. “Anorexia Nervosa: Eating Disorder or Body Image Disorder?” Australian and New Zealand Journal of Psychiatry 52, no. 1: 13–14. 10.1177/0004867417722640. 28762280

[erv70103-bib-0044] Phillipou, A. , S. L. Rossell , D. J. Castle , and C. Gurvich . 2022. “Interoceptive Awareness in Anorexia Nervosa.” Journal of Psychiatric Research 148: 84–87. 10.1016/j.jpsychires.2022.01.051. 35121272

[erv70103-bib-0045] Phillipou, A. , S. L. Rossell , C. Gurvich , D. J. Castle , N. F. Troje , and L. A. Abel . 2016. “Body Image in Anorexia Nervosa: Body Size Estimation Utilising a Biological Motion Task and Eyetracking.” European Eating Disorders Review 24, no. 2: 131–138. 10.1002/erv.2423. 26621427

[erv70103-bib-0046] Phillipou, A. , U. Schmidt , E. Neill , S. Miles , P. McGorry , and K. T. Eddy . 2025. “Anorexia Nervosa—Facts, Frustrations, and the Future.” JAMA Psychiatry 82, no. 8: 844. 10.1001/jamapsychiatry.2025.0812. 40465304

[erv70103-bib-0047] Ralph‐Nearman, C. , and R. Filik . 2020. “Development and Validation of New Figural Scales for Female Body Dissatisfaction Assessment on Two Dimensions: Thin‐Ideal and Muscularity‐Ideal.” BMC Public Health 20, no. 1: 1114. 10.1186/s12889-020-09094-6. 32677919 PMC7364654

[erv70103-bib-0048] Ralph‐Nearman, C. , A. C. Arevian , S. Moseman , et al. 2021. “Visual Mapping of Body Image Disturbance in Anorexia Nervosa Reveals Objective Markers of Illness Severity.” Scientific Reports 11, no. 1: 12262. 10.1038/s41598-021-90739-w. 34112818 PMC8192536

[erv70103-bib-0050] Rienecke, R. 2017. “Family‐Based Treatment of Eating Disorders in Adolescents: Current Insights.” Adolescent Health, Medicine and Therapeutics 8: 69–79. 10.2147/ahmt.s115775. 28615982 PMC5459462

[erv70103-bib-0051] Riva, G. , C. Malighetti , and S. Serino . 2021. “Virtual Reality in the Treatment of Eating Disorders.” Clinical Psychology & Psychotherapy 28, no. 3: 477–488. 10.1002/cpp.2622. 34048622 PMC8362149

[erv70103-bib-0052] Schmidt, U. , and J. Treasure . 2006. “Anorexia Nervosa: Valued and Visible. A Cognitive‐Interpersonal Maintenance Model and Its Implications for Research and Practice.” British Journal of Clinical Psychology 45, no. 3: 343–366. 10.1348/014466505x53902. 17147101

[erv70103-bib-0053] Shagar, P. S. , N. Harris , J. Boddy , and C. L. Donovan . 2017. “The Relationship Between Body Image Concerns and Weight‐Related Behaviours of Adolescents and Emerging Adults: A Systematic Review.” Behaviour Change 34, no. 4: 208–252. 10.1017/bec.2018.3.

[erv70103-bib-0054] Sibeoni, J. , M. Orri , S. Colin , M. Valentin , J. Pradère , and A. Revah‐Levy . 2017. “The Lived Experience of Anorexia Nervosa in Adolescence, Comparison of the Points of View of Adolescents, Parents, and Professionals: A Metasynthesis.” International Journal of Nursing Studies 65: 25–34. 10.1016/j.ijnurstu.2016.10.006. 27815986

[erv70103-bib-0055] Smeets, M. A. M. , F. Smit , G. E. M. Panhuysen , and J. D. Ingleby . 1997. “The Influence of Methodological Differences on the Outcome of Body Size Estimation Studies in Anorexia Nervosa.” British Journal of Clinical Psychology 36, no. 2: 263–277. 10.1111/j.2044-8260.1997.tb01412.x. 9167866

[erv70103-bib-0056] Steinglass, J. E. , and B. T. Walsh . 2016. “Neurobiological Model of the Persistence of Anorexia Nervosa.” Journal of Eating Disorders 4, no. 1: 19. 10.1186/s40337-016-0106-2. 27195123 PMC4870737

[erv70103-bib-0057] Steinhausen, H. C. 2002. “The Outcome of Anorexia Nervosa in the 20th Century.” American Journal of Psychiatry 159, no. 8: 1284–1293. 10.1176/appi.ajp.159.8.1284. 12153817

[erv70103-bib-0058] Stice, E. , C. N. Marti , and S. Durant . 2011. “Risk Factors for Onset of Eating Disorders: Evidence of Multiple Risk Pathways From an 8‐Year Prospective Study.” Behaviour Research and Therapy 49, no. 10: 622–627. 10.1016/j.brat.2011.06.009. 21764035 PMC4007152

[erv70103-bib-0059] Touyz, S. W. , P. J. Beumont , J. K. Collins , M. McCabe , and J. Jupp . 1984. “Body Shape Perception and Its Disturbance in Anorexia Nervosa.” British Journal of Psychiatry 144, no. 2: 167–171. 10.1192/bjp.144.2.167. 6704603

[erv70103-bib-0060] Walsh, B. T. , T. Xu , Y. Wang , E. Attia , and A. S. Kaplan . 2021. “Time Course of Relapse Following Acute Treatment for Anorexia Nervosa.” American Journal of Psychiatry 178, no. 9: 848–853. 10.1176/appi.ajp.2021.21010026. 34154394 PMC8440387

[erv70103-bib-0061] Williams, K. , J. King , and J. R. E. Fox . 2016. “Sense of Self and Anorexia Nervosa: A Grounded Theory.” Psychology and Psychotherapy: Theory, Research and Practice 89, no. 2: 211–228. 10.1111/papt.12068. 26179295

