# Supplementary material for: Examining Facets of Body Image Disturbance Across Anorexia Nervosa Illness and Recovery Stages
Source: Eur Eat Disord Rev. 2026 Apr 1;34(5):1184–92. doi: 10.1002/erv.70103 (PMC13432471; doi:10.1002/erv.70103)
Supplement: Supplementary file 1 — Figure S1: CONSORT 2025 Flow Diagram. [file ERV-34-1184-s001.docx]

**Figure 1: CONSORT 2025 Flow Diagram**



Removed After Data Cleaning (n= 933)

Repeated IP address (n= 17)

Not fitting into pre-determined participant group

categories (n= 916)

Excluded (n= 346)

Did not meet inclusion criteria (n= 203)

Did not complete all relevant measures (n= 143)

Participants Commenced Survey (n= 1,606)

Included in Data Cleaning (n= 1,260)

Included in Analysis (n= 327)
